# Neuroprotective potential of solanesol in intracerebroventricular propionic acid induced experimental model of autism: Insights from behavioral and biochemical evidence

**DOI:** 10.1016/j.toxrep.2019.10.019

**Published:** 2019-11-05

**Authors:** Ramit Sharma, Saloni Rahi, Sidharth Mehan

**Affiliations:** Department of Pharmacology, ISF College of Pharmacy, Moga, Punjab, India

**Keywords:** ETC, electron-transport chain, PPA, propionic acid, CoQ10, coenzyme-Q10, SNL, Solanesol, CNS, center nerves system, UBE3A, Ubiquitin-protein ligase E3A, MAPK3, mitogen-activated protein kinase 3, BBB, blood-brain barrier, MEM, Memantine, ARP, Aripiprazole, CTP, Citalopram, DNP, Donepezil, *i.p.*, Intraperitoneal route, ELT, escape latency, LDH, lactate dehydrogenase, AChE, acetylcholinesterase acetylcholinesterase, MDA, malondialdehyde, SOD, superoxide dismutase, NO, nitric oxide, ICV, Intracerebroventricular, *p.o.*, Oral, Autism, mitochondrial dysfunction, Coenzyme-Q10, Propionic acid, ATP, Aripiprazole, Memantine, Citalopram, Donepezil

## Abstract

Autism is the category used within the newest edition of the diagnostic and statistical manual of neurodevelopmental disorders. Autism is a spectrum of disorder where a variety of behavioural patterns observed in autistic patients, such as stereotypes and repetitive behavior, hyperexcitability, depression-like symptoms, and memory and cognitive dysfunctions. Neuropathological hallmarks that associated with autism are mitochondrial dysfunction, oxidative stress, neuroinflammation, Neuro-excitation, abnormal synapse formation, overexpression of glial cells in specific brain regions like cerebellum, cerebral cortex, amygdala, and hippocampus. ICV injection of propionic acid (PPA) (4 μl/0.26 M) mimics autistic-like behavioral and biochemical alterations in rats. Literature findings reveal that there is a link between autism neuronal mitochondrial coenzyme-Q10 (CoQ10) and ETC-complexes dysfunctions are the keys pathogenic events for autism. Therefore, in the current study, we explore the neuroprotective interventions of Solanesol (SNL) 40 and 60 mg/kg alone and in combination with standard drugs Aripiprazole (ARP) 5 mg/kg, Citalopram (CTP) 10 mg/kg, Memantine (MEM) 5 mg/kg and Donepezil (DNP) 3 mg/kg to overcome behavioral and biochemical alterations in PPA induced experimental model of Autism. Chronic treatment with SNL 60 mg/kg in combination with standard drug shows a marked improvement in locomotion, muscle coordination, long-term memory and the decrease in depressive behavior. While, chronic treatment of SNL alone and in combination with standard drug aripiprazole, citalopram, donepezil, and memantine shows the Neuroprotective potential by enhancing the cognitive deficits, biochemical alterations along with reducing the level of inflammatory mediators and oxidative stress.

## 1 Introduction

Autism is a stereotypical disorder of the center nerves system (CNS), having the involvement of communication gap, repetitive behavioral patterns and restricted interests, hyperactivity and sensory disturbances. The prevalence of autism world wide is 1:160 as per World Health Organization 2018 [1,70], while in India it is 1 in 68 children [2,37] and is very important for social welfare to accept such children with neurological disorders such as Autism. About 70 million individuals all around the world are autistic; out of which 10 million autistic patients are now in India. The social communication gap in infants for a long time converted into autism. In the comparison of infants of 6 months and children above 12 months, resulting in diminished trajectories of social behavior and losing social skills [3,68]. The communication gap seen by social responses like eye contact, smiling, crying, response, concentration and focus toward a particular object, and attention deficits [4,77]. Environmental toxins like lead, mercury, formaldehyde, pesticides and asbestos [80,78], food containing propionic acid, sodium benzoate and benzoic acid ([67,91]), genes like MAPK3 (mitogen-activated protein kinase 3) and UBE3A (ubiquitin-protein ligase E3A) [66], bacterial infection like R. Torques, and virus in the family Herpesviridae are the major etiological factors behind autism [83,48].

Propionic acid (PPA) is a short-chain fatty acid endogenous to the human body as both an intermediary of fatty acid metabolism and a metabolic end-product of enteric gut bacteria such as Clostridia and propionibacteria. PPA is a common food preservative in refined wheat and dairy products [57]. PPA, being a weak acid exists in both aqueous and lipid-soluble forms and can readily enter the systemic and CNS environments through the blood-brain barrier (BBB) via monocarboxylate transporters and can cause health- related issues if the individual cannot metabolize short-chain fatty acids adequately [88]. PPA and other short-chain fatty acids like butyrate and acetate, affect diverse physiological processes such as neurotransmitter synthesis and release and mitochondrial functioning [50].

Coenzyme-Q10 (CoQ10) is an essential compound found in every neuronal cell of the body. It is an electron carrier in the mitochondrial ETC-complexes respiratory chain, which produces about 95% of the energy for the body. Solanesol (SNL) is a natural active phytochemical isolated from Nicotiana tobacum used as a precursor for the synthesis of metabolically active Quinones, such as CoQ10. CoQ10 can change the anoxic state of the cells and tissues and has beneficial effects on the liver, brain, and heart. SNL exerts anti-oxidant, anti-bacterial, anti-inflammatory, and anti-ulcer activities. CoQ10 accepts electrons from complex-I and complex-II, transfers those electrons to complex-III to synthesize (ATP) energy and used as an electron transporter in the mitochondrial respiratory chain to perform a redox reaction for synthesizing ATP as a final product [21].

SNL restore the mitochondrial energy dysfunctions and improves the behavioral and neurochemical alterations in Huntington’s disease [52]. CoQ10 can be effective in cognitive and motor performance tests and this supplementation may improve age-related mitochondrial function Changes and mitochondria-associated with structural damage and oxidative stress [17]. CoQ10 is a powerful anti-oxidant; reducing oxidative stress and a free radical generation in neurodegenerative disorders like Alzheimer’s disease (AD), Parkinson’s disease (PD), and Autism [90,20].

CoQ10 also has membrane-stabilizing properties and its insufficiency mediate the ETC-complex-I, II, III dysregulation and cause neuronal mitochondrial intervention in Autism such as the production of ROS, RNS, neuro excitation by increased glutamate, glial cells over activation mediated neuroinflammation, neurotransmitter imbalance, and cholinergic dysfunction [31].

There are a variety of approachable drug therapies used clinically to provide symptomatic relief in autistic patients, such as aripiprazole (ARP) [63], memantine (MEM) [18], donepezil (DNP) [34] and citalopram (CTP) [44] in attenuation of behavioral alterations.

Dysfunction in the efficiency of mitochondrial CoQ10 responsible for the neuropathological cascade that will eventually cause autism. SNL as a precursor of CoQ10 can improve the progression of the disease and improve the behavioral and neurochemical characteristics of autism-like neuroinflammation, neuroexcitation, neurotransmitter imbalance, and oxidative stress.

Current drug therapy used only to provide symptomatic relief. Clinicians approach Aripiprazole; for hyperexcitability, Citalopram; for depression-like symptoms, Memantine; for irritating behaviour, and Donepezil used for memory and cognitive dysfunctions, clinically in autistic patients. Aripiprazole is D2 and 5HT1A receptor partial agonist, having a clinical dose of 2 mg/kg (p.o.), 5 mg/kg (p.o.), 15 mg/kg (p.o.), 30 mg/kg (p.o.) [[11], [12], [13], [14], [15], [16], [17], [18], [19], [20], [21], [22], [23], [24], [25], [26]] and pre-clinical dose of 1 mg/kg (p.o.), 1.5 mg/kg (i.p.), 10 mg/kg (*i.p*.) [29,37,59,61]. Citalopram is SSRI used to treat depression-like symptoms at clinical dose of 2.5 mg (*p.o*.), 10 mg (*p.o*.), 20 mg (*p.o*.),60 mg (*p.o*.) [69–7] and pre-clinical dose of 1 mg/kg, 5 mg/kg, 10 mg/kg, 20 mg/kg (s.c.) ([75–54], [60]). Memantine is an NMDA-Glutamate receptor antagonist and decrease Ca2+ influx reduce the irritative behaviour at a clinical dose of 3 mg (*p.o*.), 5 mg (*p.o*.), 10 mg (*p.o*.), 15 mg (*p.o*.), 20 mg (*p.o*.) ([86], [96–25], [46]) and pre-clinical dose of 30 mg/kg (*p.o*), 2.5 mg/kg,5 mg/kg,10 mg/kg, 20 mg/kg, (*i.p*.) [[53], [54], [55], [56], [57], [58], [59], [60], [61], [62], [63], [64]]. Donepezil used in autism to reduce the memory and cognitive dysfunctions at clinical doses of 5-10 mg/kg (*p.o*.),1.5-2.5 mg/kg (*p.o*.), 2.5 mg/kg (*p.o*.) [[6], [7], [8], [9], [10], [11], [12], [13], [14], [15], [16]] and at pre-clinical dose of 3 mg/kg (p.o.), 6-8 mg/kg (p.o), 0.3 mg/kg, (i.p.), 0.3 mg/kg (s.c.). [[43], [44], [45], [46], [47], [48], [49], [50], [51], [52], [53], [54], [55], [56], [57], [58]].

Therefore, the current study explored the neuroprotective effect of chronic administration of SNL as a neuroprotective agent by administering alone and in combination with standard drugs Aripiprazole (ARP), memantine (MEM), donepezil (DNP) and citalopram (CTP) in ICV-PPA induces autistic rats.

## 2 EXPERIMENTAL ANIMALS

Wistar rats (weighing 250-300 g, aged 4-6 months, either sex) got from the Central Animal House, ISF College of Pharmacy, Moga, Punjab. The animals kept in polyacrylic cages with a wire mesh top and soft bedding. They kept under standard husbandry conditions of 12 hour light and dark cycle with food and water ad libitum maintained at 23 2 The experimental protocol approved by the Institutional Animal Ethics Committee (IAEC) with registration no. 816/PO/ReBiBt/S/04/CPCSEA as per the guidelines of the Committee for the purpose of control and supervision of experiments on animals, Government of India (ISFCP/IAEC/CPCSEA/Meeting No.23/2018/ Protocol No. 378). Animals acclimatized to laboratory conditions before experimentation.

### 2.1 Drugs and chemicals

Propionic acid (PPA) purchased from Sigma–Aldrich (USA). Solanesol (SNL) provided as an ex gratia sample from BAPEX, India. Standard drugs like Aripiprazole (ARP), Citalopram (CTP), Donepezil (DNP) and Memantine (MEM) provided as an ex gratia sample.

All other chemicals used in the study are of analytical grade. Solutions of the drugs and chemicals freshly prepared before use.

Solanesol (SNL) dissolved in water with 2% ethanol administered by the oral route (p.o.). [52] Aripiprazole (ARP) dissolved in DMSO [89], Citalopram (CTP) dissolved in Saline [41], Donepezil (DNP) dissolved in phosphate buffer (pH 7.4) [61], Memantine (MEM) dissolved in Saline [45] they all administered by intraperitoneal route (i.p.)

The total animals used in this study were 59 animals, and each group comprises 7 animals. Chronic administration of ICV-PPA used for induced Autism. Chronic administration of ICV-PPA given from day 1 st to 11th of protocol schedule study, whereas the protocol drug SNL and standard drugs ARP, CTP, DNP, MEM started on 12th day and continuous up to 44th day.

**Fig. 1:**
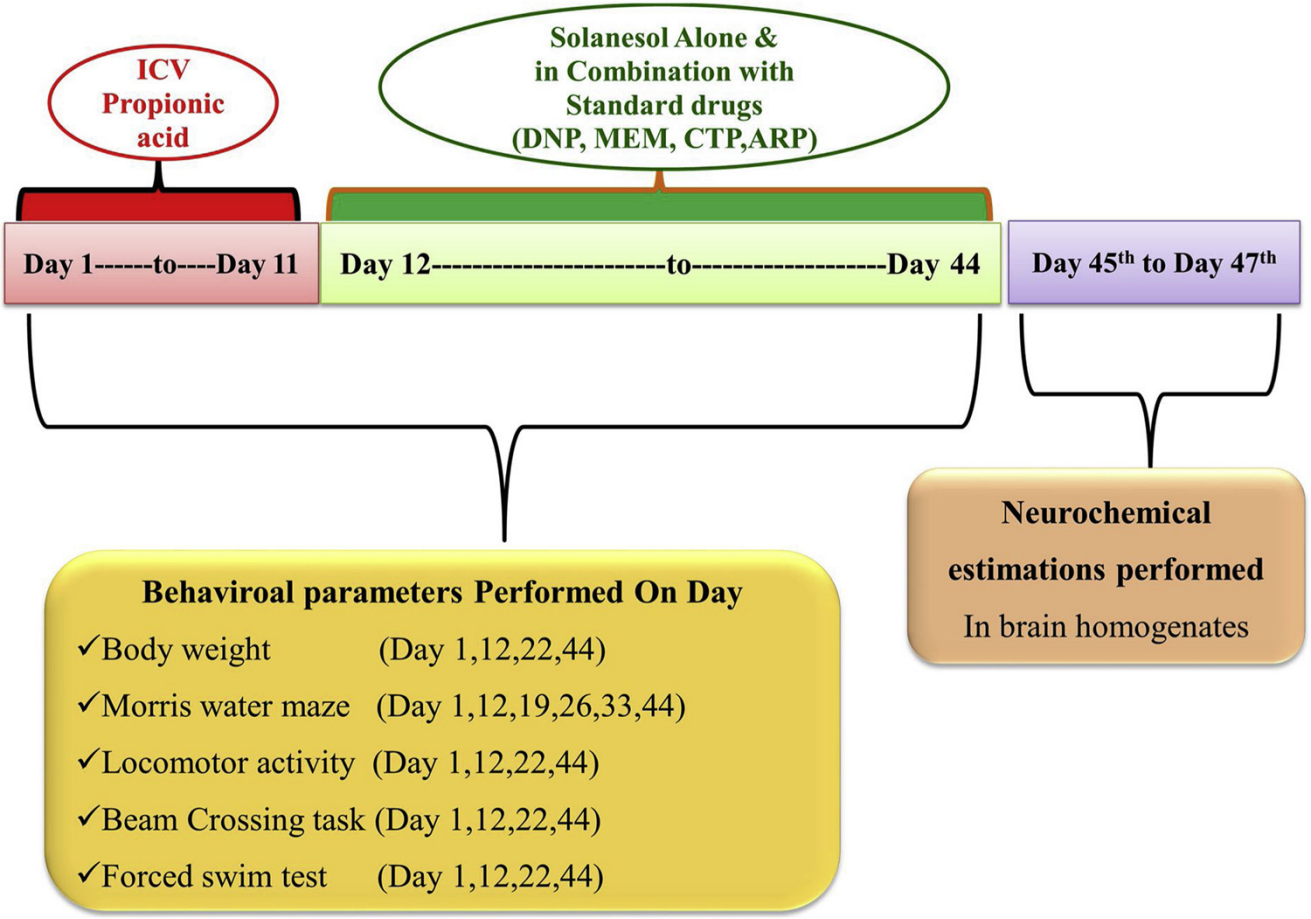
Experimental protocol schedule (Behavioral & Biochemical estimations).

On specific days, behavioral parameters like Morris water maze, locomotor activity, beam crossing task and forced swim test were performed. After the 44th day of the protocol, the animals made to sacrifice and brains were isolated to perform biochemical, inflammatory, and neurochemical estimation (Table 1, Fig. 1).

**Table 1:** Treatment Groups.

S.no.	Group	Drug treatment
1.	Sham Control	No treatment
2.	Solanesol perse	60 mg/kg (*p.o.*) from day 12 to 44
3.	Propionic acid	0.26 M/4 μl/unilateral (ICV) from day 1 to 11
4.	Propionic acid + Solanesol	0.26 M/4 μl/unilateral (ICV) from day 1 to 11 + 40 mg/kg (*p.o.*) from day 12 to 44
5.	Propionic acid + Solanesol	0.26 M/4 μl/unilateral (ICV) from day 1 to 11 + 60 mg/kg (*p.o.*) from day 12 to 44
6.	Propionic acid + Solanesol + Aripiprazole	0.26 M/4 μl/unilateral (ICV) from day 1 to 11 + 40 mg/kg (*p.o.*) from day 12 to 44 + 5 mg/kg (*i.p.*) from day 12 to 44
7.	Propionic acid + Solanesol + Donepezil	0.26 M/4 μl/unilateral (ICV) from day 1 to 11 + 40 mg/kg (*p.o.*) from day 12 to 44 + 3 mg/kg (*i.p.*) from day 12 to 44
8.	Propionic acid + Solanesol + Citalopram	0.26 M/4 μl/unilateral (ICV) from day 1 to 11 + 40 mg/kg (*p.o.*) from day 12 to 44 + 10 mg/kg (*i.p.*) from day 12 to 44
9.	Propionic acid + Solanesol +Memantine	0.26 M/4 μl/unilateral (ICV) from day 1 to 11 + 40 mg/kg (*p.o.*) from day 12 to 44 + (*i.p.*) from day 12 to 44 + 5 mg/kg

#### 2.1.1 Experimental animal model of icv-ppa induced autism in rats

Rats acclimatized to the laboratory conditions. After acclimatization, rats anesthetized with an intraperitoneal injection of ketamine (75 mg/kg**).** The body of the anesthetized rat placed on a warm pad with the head positioned in the stereotaxic frame (Stoelting Co., Wood Dale, IL, USA). The head, fixed with the help of the incisor bar and ear bars in a symmetrical position. Before the initiation of the surgical procedure, the position of the head corrected such that the coordinates of bregma and lambda matched and were at the same level. The head of rats shaved, the scalp cleaned with 70% ethanol and incised with the scalpel (mid-sagittal), skin retracted and the skull exposed to locate bregma and lambda which marked to facilitate the determination of the coordinates for ICV injection Cotton swabs dipped in normal saline, put on rat eyes to prevent dehydration and cotton buds were used to cease bleeding. A hole drilled through the parietal bone to access a right lateral cerebral ventricle (Stereotaxic coordinates: anteroposterior from bregma 1.3 mm, mediolateral from mid-sagittal suture 1.8 mm, dorso- ventral from the skull 3.0 mm) and cannula of 2.5 cm length inserted in the burr hole and the cannula locked using a plastic ear-pin. The hole repaired with dental cement and a surgical incision, then sutured with absorbable surgical suture attached to the sterile surgical needle. On day 1 st to 11th PPA (4.0 μL of a 0.26 M solution injected through a hypodermic needle of 0.4 mm external diameter attached to a 10-μl Hamilton microliter syringe in the left lateral cerebral ventricle over 10 minutes duration (1 μl/min). After injection, the Hamilton® microneedle not displaced for 5 minutes to facilitate the diffusivity of drugs in CSF [87].

For post-operative care, rats housed individually in polyacrylic cages containing warm cloth and husk removed and special care performed until they regained spontaneous movement i.e. Approximately 2–3 hours after anesthesia [5]. The room temperature maintained at 25 ± 3 °C. For 2–3 days, milk and glucose water kept in the cages to avoid any physical trauma after surgery. Rats, intraperitoneally injected for 3 days with gentamycin (35 mg/kg) to avert sepsis, and lignocaine gel applied to the sutured area to circumvent pain, and Neosporin powder, sprinkled to prevent bacterial skin infections. Clinical signs monitored regularly after the surgery, including general body condition and dehydration. After 7 days, proper diet and water intake started by rats and regained spontaneous movement showing signs of recovery [49].

### 2.2 Parameters evaluated

#### 2.2.1 Measurement of body weight

Bodyweight noted on the day 1 st, day 12th, day 22nd and day 44th of the experiment [19].

#### 2.2.2 Behavioral parameters

##### 2.2.2.1 Morris water maze task

Spatial learning and memory of animals tested in a Morris water maze on day 1 st, 12th, 19th, 26th, 33rd & 44th day of protocol schedule in a circular water tank filled with water (25 ± 1 °C) to a depth of 40 cm and hidden platform submerged down 2 cm in water [56]. Acquisition phase includes training sessions of 4 trials per session (once from each starting point) for 5 days day 1 st, 12th, 19th, 26th, and 33rd and escape latency (ELT) to reach the hidden platform noted having the ceiling time of 120 s. If the rat did not locate the hidden platform within the maximum time of 120 s, it gently placed on the platform and allowed to remain there for 20 seconds. After the acquisition phase, a probe test on day 44 conducted by removing the platform. Rats allowed to swim freely in the pool for 120 s and TSTQ (time spend in target quadrant) recorded. TSTQ showed memory consolidation that had taken place after learning [30]

##### 2.2.2.2 Spontaneous Locomotor activity

Animals tested for locomotor activity by using an actophotometer on day 1 st, 12th, 33th, and 44th. Each animal observed for 5 min in a square closed arena equipped with infrared light-sensitive photocells using a digital actophotometer and values expressed as counts per 5 min [69,82].

##### 2.2.2.3 Beam crossing task

Each animal tested for its motor coordination ability on days 1, 12th, 33th, and 44th. The number of slips in each trial recorded, and the motor performance of rats scored on a scale ranging from 0 to 4. A score of 0 assigned to the animal that could readily transverse the beam. Score 1, 2 and 3 given to animals showed mild, moderate, and severe impairment, respectively. Score 4 assigned to the animals unable to walk on the beam [81].

##### 2.2.2.4 Forced swim test

Forced swim test performed on day 1 st, 12th, 33th, and 44th with the purpose to evaluate the depressive behavior of rats. During the training period, the first exposure of rats in the tank is for 15 minutes and the second performed 24 hours after the first, with an exposure period of 5 minutes. The testing period for rat comprises a single 6- minute exposure, with the first 2 minutes serving as a habituation period and the last 4 minutes comprising the test itself, which yields the duration of immobility [38].

#### 2.2.3 Estimation of biochemical parameters

##### 2.2.3.1 Preparation of Brain Homogenate

On the 45th day of protocol schedule, animals sacrificed by decapitation; brains removed and washed with ice-cold isotonic saline solution. Brain samples, then homogenized with 10 times (w/v) ice-cold 0.1 M phosphate buffer (7.4). The homogenate centrifuged at 10,000 g for 15 min, supernatant separated and aliquots used for biochemical estimation [40].

#### 2.2.4 Estimation of mitochondrial ETC-Complex enzymes activity

##### 2.2.4.1 Mitochondrial ETC Complex-I enzyme (NADPH dehydrogenase) activity

Complex-I enzyme activity measured spectrophotometrically at 37 °C by the rate of NADH oxidation at 340 nm in an assay medium for a duration of 3 min. Reactions performed in the absence and the presence of 2 mM rotenone and the rotenone-sensitive activity attributed to complex-I [35].

##### 2.2.4.2 Mitochondrial ETC Complex-II enzyme (Succinate dehydrogenase/SDH) activity

Complex-I enzyme activity measured spectrophotometrically at 490 nm. Sodium Succinate solution mixed with 50 μl of a gradient fraction of homogenate. Results expressed as INT reduced μmol/mg protein [10,74].

##### 2.2.4.3 Mitochondrial ETC Complex-V enzyme activity (ATP)

Aliquots of homogenates sonicated immediately in ice-cold Perchloric acid (0.1 N) to inactivate ATPases. After centrifugation (14.000 g, 4 °C and 5 min) supernatants containing ATP neutralized with 1 N NaOH and stored at −80 °C until analysis. ATP levels in supernatants quantified using a reverse-phase HPLC (Perkin Elmer). The detection wavelength was 254 nm and the reference solution of ATP prepared according to the dissolving standard. Results expressed as nM/mg protein [71].

##### 2.2.4.4 Mitochondrial ETC-CoQ10 activity

CoQ10 from brain tissues and mitochondria extracted and measured by HPLC as described by Mitochondria homogenized using micro glass tissue grinder in 0.5 ml potassium phosphate buffer pH 7.4 containing 1 mM dithiothreitol and 2 ml of ethanol containing 1 μg/ml butylated hydroxytoluene added and ubiquinone extracted with 5 ml hexane. After vigorous shaking, 4 ml of the hexane layer dried under N2. The residue dissolved in 100 μl ethanol containing BHT and subjected to HPLC analysis. CoQ10 quantified using the C18 column with a mobile phase methanol: hexane (90:10 v/v), detected at a wavelength of 275 nm and results expressed as nM/g protein [84].

#### 2.2.5 Estimation of neurotransmitters

##### 2.2.5.1 Assessment of glutamate

Glutamate quantified after the derivatization with o-phthalaldehyde/ β-mercaptoethanol (OPA/β-ME) and the quantitative analysis of a tissue sample performed and results expressed as mg/mg protein [39].

##### 2.2.5.2 Estimation of Dopamine

Dopamine levels in the brain estimated using high- performance liquid chromatography (HPLC) using an electrochemical detector (ECD). The mobile phase comprising sodium citrate buffer (pH 4.5)–acetonitrile (87:13, v/v). The sodium citrate buffer comprised 10 mM citric acid, 25 mM NaH2HPO4, 25 mM EDTA (ethylene diamine tetraacetic acid), and 2 mM 1-heptane sulfonic acid. The electrochemical conditions of the experiment were +0.75 V, with sensitivity ranging from 5 to 50 nA. The separation carried out at a flow rate of 0.8 ml/min. The samples (20 μl) injected manually. Brain samples homogenized in a homogenizing solution containing 0.2 M Perchloric acid and centrifuged at 12,000 g for 5 min. Results expressed as mg/mg protein [65].

##### 2.2.5.3 Estimation of Acetylcholine (Ach)

A diagnostic kit (Krishgen diagnostics, India) was used to measure acetylcholine. Samples and all the reagents were prepared as described in the kit. The optical density of the reaction mixture determined at 540 nm in the microtiter plate. Results are expressed as ng/mg protein [73].

#### 2.2.6 Estimation of neuroinflammatory biomarkers

##### 2.2.6.1 Estimation of TNF-α levels

A diagnostic kit (Krishgen diagnostics, India) was used to measure TNF-α. Samples and all the reagents were prepared as described in the kit. The optical density of the reaction mixture determined at 450 nm in the microtiter plate and results expressed as pg/mg protein [94].

##### 2.2.6.2 Estimation of IL-1β levels

A diagnostic kit (Krishgen diagnostics, India) was used to measure IL-1β. Samples and all the reagents were prepared as described in the kit. The optical density of the reaction mixture determined at 450 nm in the microtiter plate and results expressed as pg/mg protein [92].

##### 2.2.6.3 Protein estimation

The protein content measured by using the Coral protein estimation kit (Biuret method) [72].

##### 2.2.6.4 Estimation of lactate dehydrogenase (LDH) assay

A diagnostic kit (Coral Diagnostics, India) was used to measure lactate dehydrogenase activity in rat brain homogenate and expressed as IU/L [22].

##### 2.2.6.5 Estimation of acetylcholinesterase (AChE) levels

The quantitative measurement of acetylcholinesterase activity in the brain performed according to the method described by The assay mixture contained 0.05 ml of supernatant, 3 ml of 0.01 M sodium phosphate buffer (pH 8), 0.10 ml of acetylthiocholine iodide and 0.10 ml of DTNB (Ellman reagent). The change in absorbance measured immediately at 412 nm spectrophotometrically. The enzymatic activity in the supernatant expressed as protein [79].

##### 2.2.6.6 Measurement of reduced glutathione levels

Reduced glutathione in the brain estimated according to the method described by [23]. 1 ml supernatant precipitated with 1 ml of 4% sulfosalicylic acid and cold digested at 4 °C for 1 h. The samples centrifuged at 1200×g for 15 min. To 1 ml of the supernatant, 2.7 ml of phosphate buffer (0.1 M, pH 8) and 0.2 ml of 5,5′-dithiobis-(2- nitrobenzoic acid) (DTNB) added. The yellow color that develops to measure immediately at 412 nm using a spectrophotometer. The concentration of glutathione in the supernatant expressed as μM/mg protein [22].

##### 2.2.6.7 Estimation of malondialdehyde (MDA) levels

The quantitative measurement of Malondialdehyde (MDA) end product of lipid peroxidation–in brain homogenate performed according to the method of [12] the amount of MDA measured, after its reaction with thiobarbituric acid, at 532 nm using a spectrophotometer. The concentration of MDA expressed as nM/mg protein [32].

##### 2.2.6.8 Estimation of superoxide dismutase (SOD) activity

The SOD activity measured according to the method described by following spectrophotometrically the auto-oxidation of epinephrine at pH 10.4. In this method, supernatant (0.2 ml) of the brain homogenate was mixed with 0.8 ml 50 mM glycine buffer, pH 10.4 and the addition of 0.02 ml of epinephrine started the reaction. After 5 minutes, the absorbance measured spectrophotometrically at 480 nm. The activity of SOD expressed as nM/mg protein [42].

##### 2.2.6.9 Assessment of nitrite levels

The accumulation of nitrite in the supernatant, an indicator of the production of nitric oxide (NO), determined by a colorimetric assay using Greiss reagent (0.1% N-(1-naphthyl) ethylenediamine dihydrochloride, 1% sulfanilamide and 2.5% phosphoric acid) as described by. Equal volumes of supernatant and Greiss reagent mixed, the mixture incubated for 10 min at room temperature in the dark and the absorbance determined at 540 nm spectrophotometrically. The concentration of nitrite in the supernatant determined from a sodium nitrite standard curve and expressed as μM/mg protein [9].

### 2.3 Statistical analysis

All the results are expressed as mean and standard error mean (SEM). Data analyzed using two ways ANOVA followed by Post hoc test Bonferroni multiple comparison test and one way ANOVA followed by Post hoc test Tukey’s multi comparison test.

## 3 Result and discussion

### 3.1 Results

#### 3.1.1 Neuroprotective effect of SNL on body weight in ICV-PPA treated autistic rats

**Fig. 2:**
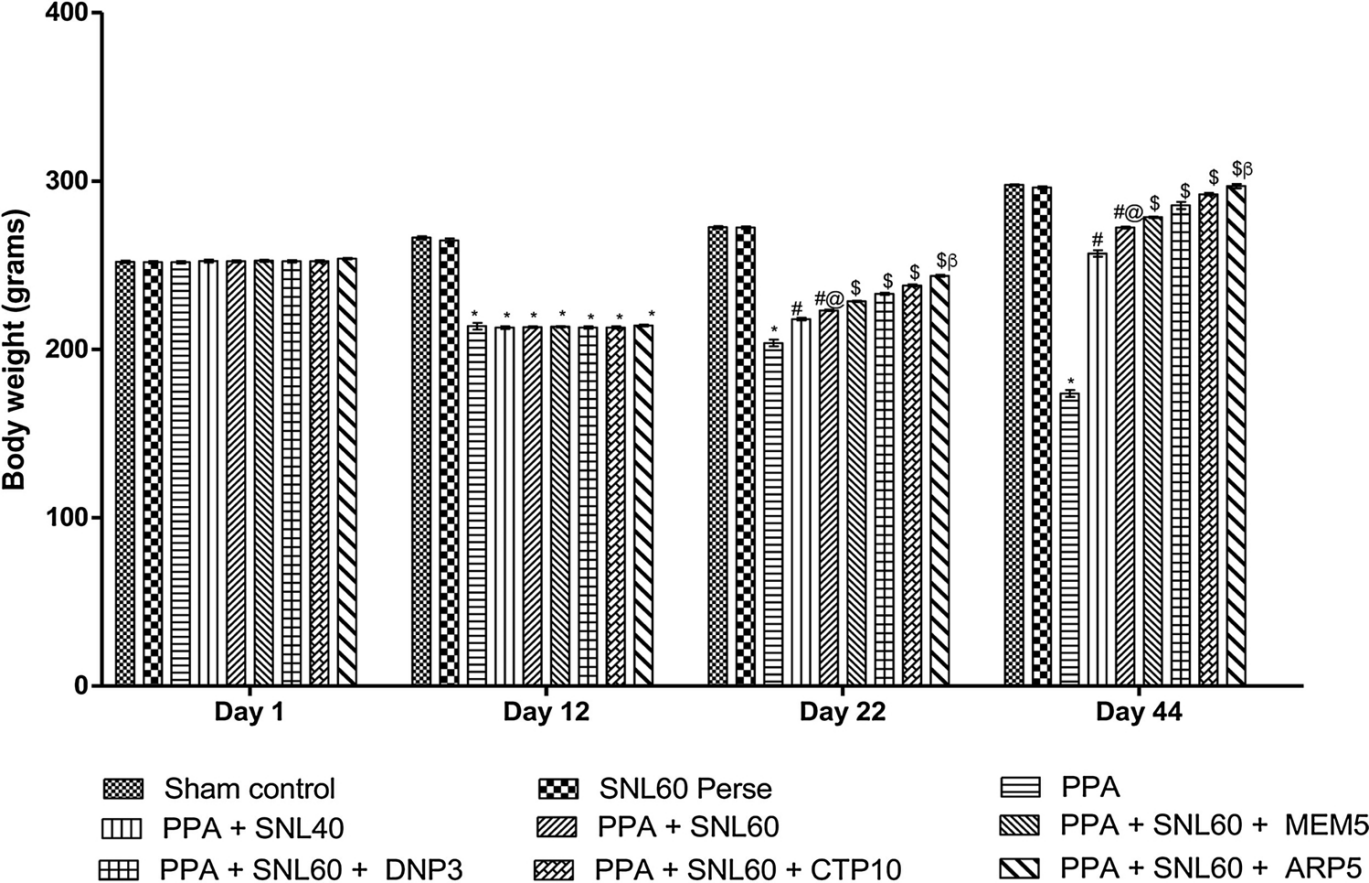
Neuroprotective effect of SNL on body weight in ICV-PPA treated autistic rats. Values expressed as mean ± SEM; * p < 0.001 versus sham control and SNL60 *perse*; **#** p < 0.001versus PPA; **#@** p < 0.001 versus PPA + SNL40; $ p < 0.001 versus PPA  + SNL60; $β p < 0.001 versus CTP10, DNP3, MEM5 (two way ANOVA followed by Bonferroni’s multiple comparison test)

Body weight measured on day 1 st, 12th, 22nd, and 44th. ICV-PPA treated rats showed a gradual decrease in body weight during administration. A significant decrease in the weight recorded at the end of the protocol schedule (p < 0.001). Chronic treatment with SNL 40 and 60 mg/kg alone and in combination with standard drugs MEM 5 mg/kg, DNP 3 mg/kg, CTP 10 mg/kg and ARP 5 mg/kg significantly and dose-dependently on day 22th restored the body weight loss as compared to ICV-PPA treated group (p < 0.001). Among these, SNL40 and 60 mg/kg in combination with ARP found to be more effective in the restoration of body weight on days 22nd and 44th (Fig. 2).

### 3.2 Behavioral parameters

#### 3.2.1 Neuroprotective effect of SNL in spatial memory using Morris water maze task in ICV-PPA treated autistic rat

**Fig. 3:**
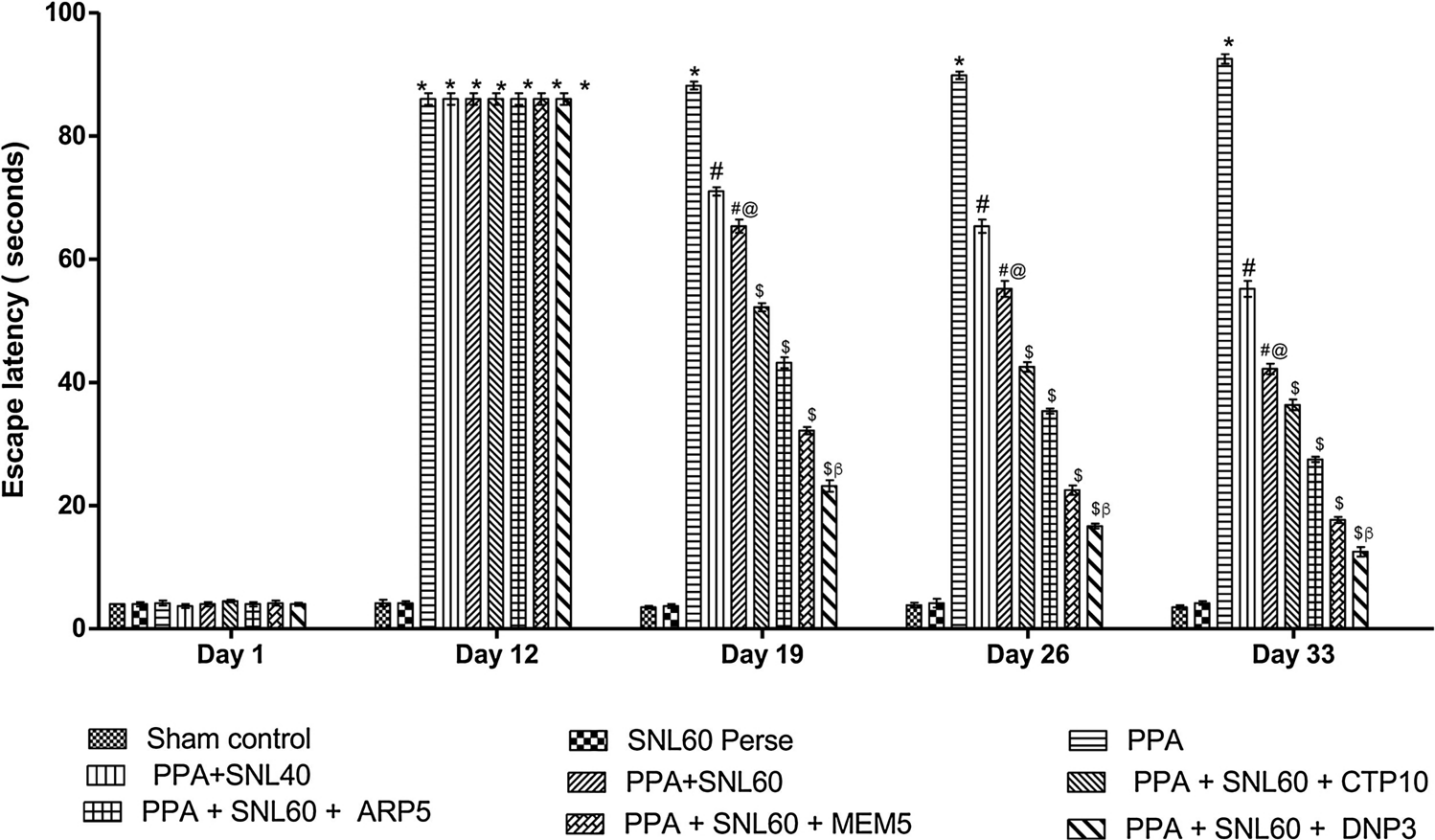
Neuroprotective effect of SNL in spatial memory using Morris water maze task in ICV- PPA treated autistic rat. Values expressed as mean ± SEM; * p < 0.001 versus sham control and SNL60 *perse*; **#** p < 0.001versus PPA; **#@** p < 0.001 versus PPA + SNL40; $ p < 0.001 versus PPA  + SNL60; $β p < 0.001 versus MEM5, ARP5, CTP10 (two way ANOVA followed by Bonferroni’s multiple comparison test)

**Fig. 4:**
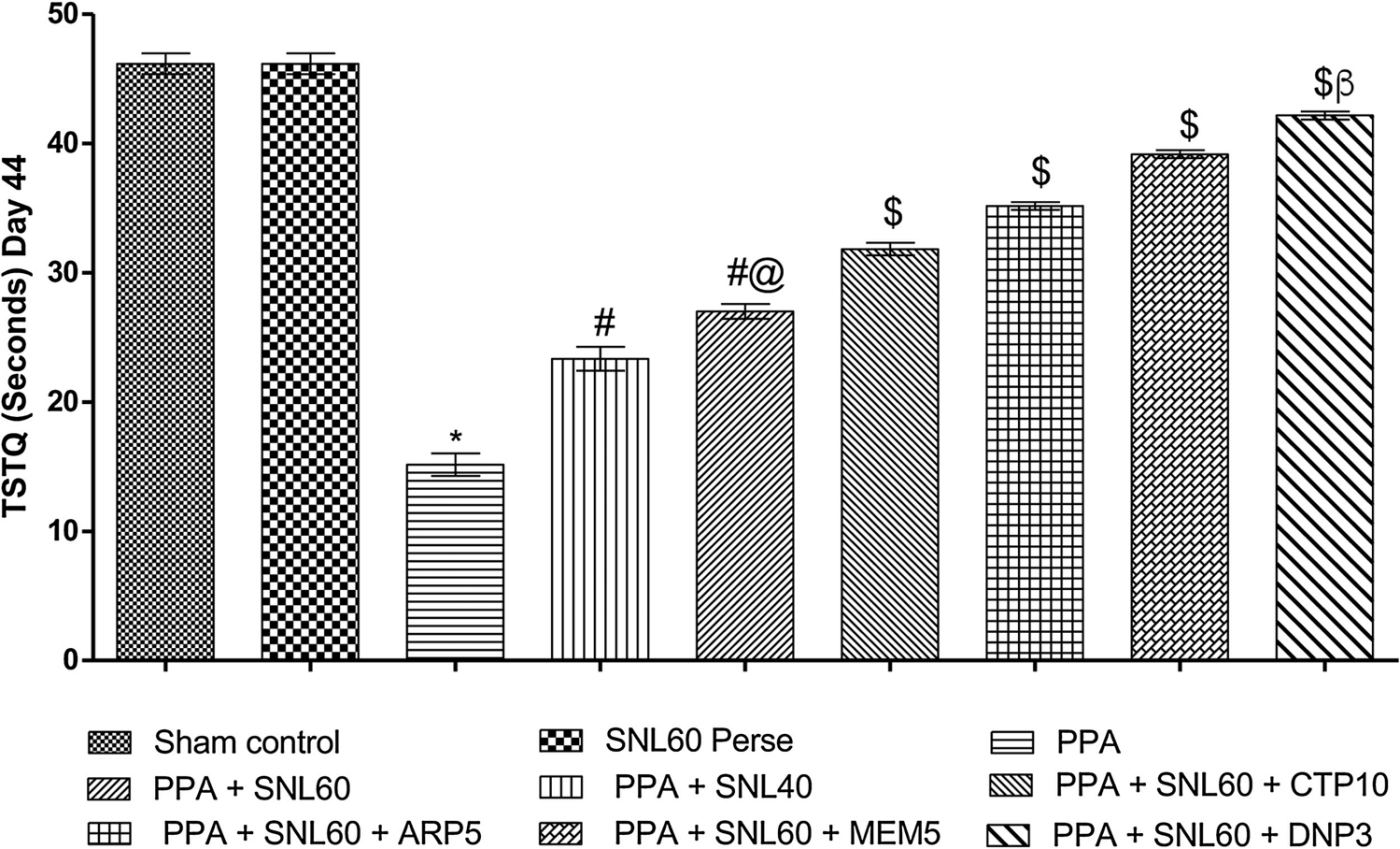
Neuroprotective effect of SNL in TSTQ using morris water maze task in ICV-PPA treated autistic rat. Values expressed as mean ± SEM; * p < 0.001 versus sham control and SNL60 *perse*; **#** p < 0.001versus PPA; **#@** p < 0.001 versus PPA + SNL40; $ p < 0.001 versus PPA  + SNL60; $β p < 0.001 versus MEM5, ARP5, CTP10 (two way ANOVA followed by Bonferroni’s multiple comparison test)

As per the protocol schedule, ELT (escape latency time) observed on day 1 st, 12th, 19th, 26th, and 33rd and on day 44th TSTQ noted. Based on the observations, long term administered ICV-PPA treated rats showed a gradual increase in ELT and a decrease in TSTQ during administration. A significant decrease in TSTQ and an increase in ELT (p < 0.001) on day 19th and continue to decrease on day 26th, 33rd, and 44th. Chronic treatment with SNL 40 mg/kg and 60 mg/kg alone and in combination with standard drugs MEM 5 mg/kg, DNP 3 mg/kg, CTP 10 mg/kg and ARP 5 mg/kg, significantly and dose-dependently restored increase in ELT and increase TSTQ as compared with ICV-PPA treated group (p < 0.001). The effective restoration observed in SNL 40 mg/kg and 60 mg/kg in combination with DNP group which shows an improvement in long-term memory (Fig. 3, Fig. 4).

#### 3.2.2 Neuroprotective Effect of SNL on locomotion activity using actophotometer in

ICV-PPA induced autistic rat

**Fig. 5:**
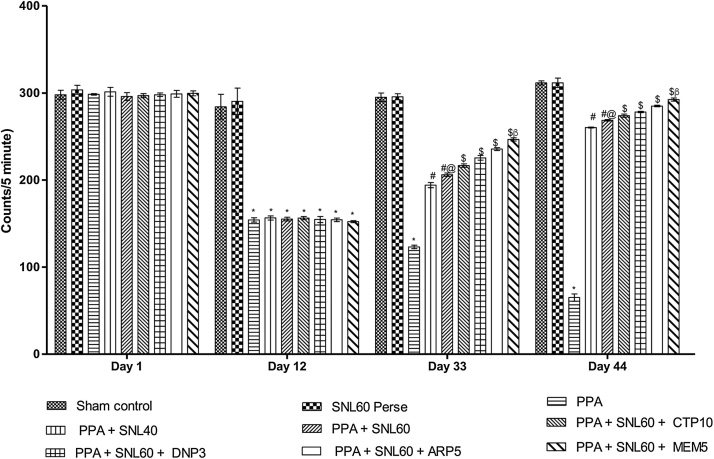
Neuroprotective Effect of SNL on locomotion activity using actophotometer in ICV-PPA induced autistic rat. Values expressed as mean ± SEM; * p < 0.001 versus sham control and SNL60 *perse*; **#** p < 0.001versus PPA; **#@** p < 0.001 versus PPA + SNL40; $ p < 0.001 versus PPA  + SNL60; $β p < 0.001 versus ARP5, DNP3, CTP10 (two way ANOVA followed by Bonferroni’s multiple comparison test).

As per the protocol schedule, the locomotor activity observed by using an actophotometer on day 1 st, 12th, 33th, and 44th. On day 1 st there was no significant difference found between all the treatment groups. On day 12th rats treated with ICV-PPA showed a significant decrease in ambulatory movements as compared to sham and SLN 60 mg/kg per se group (p < 0.001). Long-term administration of SNL 40 and 60 mg/kg alone and in combination with standard drugs MEM 5 mg/kg, DNP 3 mg/kg, CTP 10 mg/kg and ARP 5 mg/kg shows an increase in the locomotor activity when compared with ICV-PPA treated group (p < 0.001). Among the selected doses, SNL 40 and 60 mg/kg along with MEM5 showed significantly dose-dependent increase in locomotor activity of rats (Fig. 5)

#### 3.2.3 Neuroprotective Effect of SNL on muscle coordination using beam crossing task in ICV-PPA treated autistic rats

**Fig. 6:**
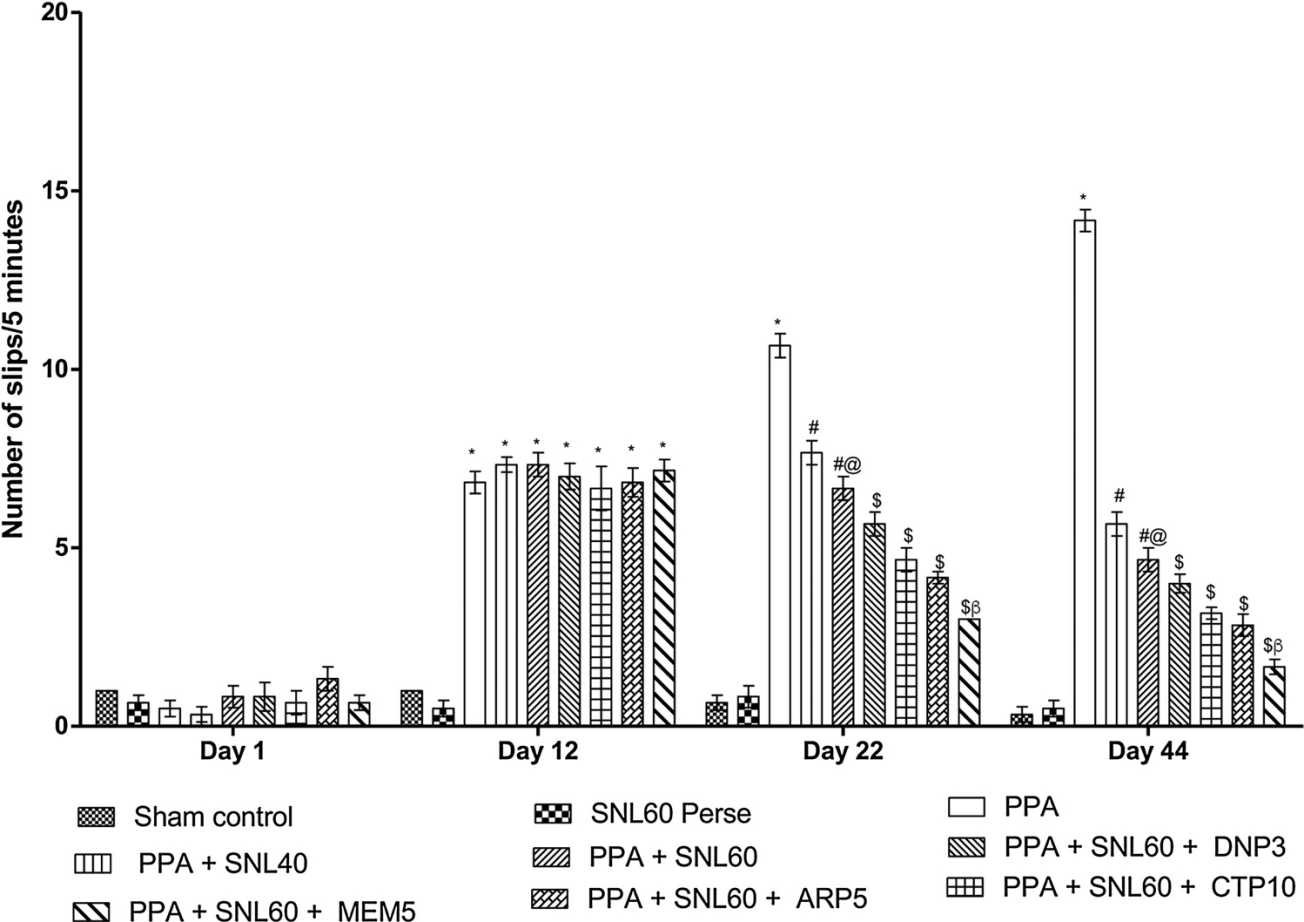
Neuroprotective Effect of SNL on muscle coordination using a beam crossing task in ICV-PPA treated autistic rats. Values expressed as mean ± SEM; * p < 0.001 versus sham control and SNL60 *perse*; **#** p < 0.001versus PPA; **#@** p < 0.001 versus PPA + SNL40; $ p < 0.001 versus PPA  + SNL60; $β p < 0.001 versus ARP5, MEM5, DNP3 (two way ANOVA followed by Bonferroni’s multiple comparison test)

The beam crossing task performed on days 1 st, 12th, 22nd, and 44th to observe muscle coordination. There was no significant difference found between all the treatment groups on day 1 st. The rats treated with ICV-PPA showed a significant increase in the number of slips (p < 0.001) on day 12th. On day 22nd and 44th chronic treatment with SNL 40 mg/kg and 60 mg/kg (p.o) alone and in combination with standard drugs MEM 5 mg/kg, DNP 3 mg/kg, CTP 10 mg/kg and ARP 5 mg/kg dose-dependently decrease the number of slips as compared to ICV-PPA treated group (p < 0.001). There was an efficient decrease in the number of slips when SNL 40 mg/kg and 60 mg/kg (p.o) combined with CTL 10 mg/kg given, decreases the number of slips and improves balance beam-walking performance (Fig. 6).

#### 3.2.4 Neuroprotective Effect of SNL on immobility phase using a forced swim test in ICV-PPA treated autistic rats

**Fig. 7:**
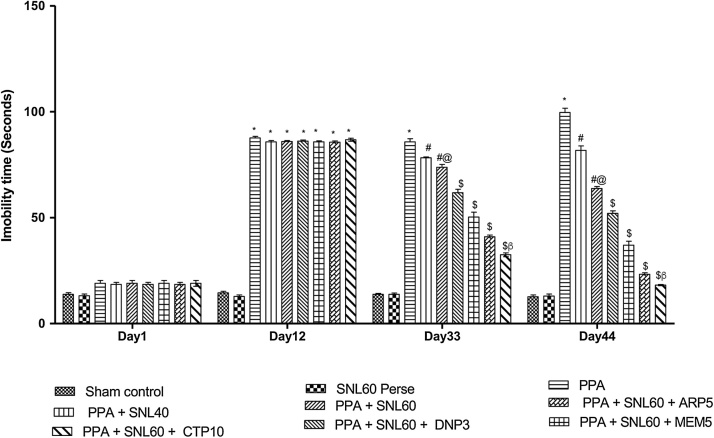
Neuroprotective Effect of SNL on immobility phase using Forced swim test in ICV-PPA treated autistic rats. Values expressed as mean ± SEM; * p < 0.001 versus sham control and SNL60 *perse*; **#** p < 0.001versus PPA; **#@** p < 0.001 versus PPA + SNL40; $ p < 0.001 versus PPA  + SNL60; $β p < 0.001 versus DNP3, CTP10, ARP5 (two way ANOVA followed by Bonferroni’s multiple comparison test).

As per the protocol schedule, immobility time observed using a force swim test on day 1 st, 12th, 33th, and 44th. On day 1 st of the observation, there was no significant difference found between all the treatment groups. The chronic administration of ICV-PPA in rats showed a significant increase in immobility time (p < 0.001) which recorded at the end of the protocol schedule. On day 12th a significant increase in the immobility time observed. The decrease in immobility seen during chronic treatment with SNL 40 mg/kg and 60 mg/kg (p.o) alone and in combination with standard drugs MEM 5 mg/kg, DNP 3 mg/kg, CTP 10 mg/kg and ARP 5 mg/kg (p < 0.001) on day 33rd and 44th. Pre-treatment with SNL 40 mg/kg and 60 mg/kg (p.o) along with MEM5 significantly and dose-dependently decreases the immobility time and found to be more effective in regaining mobility exhibiting a loss in depressive behavior (Fig. 7).

### 3.3 Biochemical estimations

#### 3.3.1 Neuroprotective effect of SNL in mitochondrial ETC complexes enzyme level in ICV-PPA treated autistic rats

The chronic administration of neurotoxin PPA in rats showed a significant decrease (p < 0.001) in mitochondrial complex I, II, V and CoQ10 activity in brain homogenate. Chronic treatment with SNL 40 and 60 mg/kg alone and in combination with standard drugs MEM 5 mg/kg, DNP 3 mg/kg, CTP 10 mg/kg and ARP 5 mg/kg remarkably restore above complexes and CoQ10 activity. The influential restoration is seen in SNL 40 and 60 mg/kg along with ARP 5 mg/kg (Table 2).

**Table 2:** Neuroprotective effect of SNL in mitochondrial ETC complexes enzyme level in ICV-PPA treated autistic rats.

Grouping	Mitochondrial complexes enzyme level			
	Complex-I (nM/mg protein)	Complex -II (nM/mg protein)	Complex -V (nM/mg protein)	CoQ_10_ (nM/g protein)
Sham control	11.85 ± 0.61	8.46 ± 0.25	588.0 ± 2.08	7.28 ± 0.06
SNL *perse*	11.25 ± 0.40	8.38 ± 0.23	588.0 ± 2.44	7.38 ± 0.15
PPA 4 μl/0.26 M	2.76 ± 0.36*	1.52 ± 0.0*	130.3 ± 16.97*	0.83 ± 0.09*
PPA + SNL40	3.58 ± 0.37^#^	1.73 ± 0.07^#^	250.3 ± 2.51^#^	1.91 ± 0.23^#^
PPA + SNL60	4.35 ± 0.42^#@^	2.11 ± 0.18^#@^	329.7 ± 4.90^#@^	2.51 ± 0.15^#@^
PPA + SNL60+ CTP10	5.53 ± 0.22^$^	3.45 ± 0.11^$^	406.7 ± 8.49 ^$^	3.90 ± 0.24^$^
PPA + SNL60+MEM5	6.03 ± 0.30^$^	4.04 ± 0.12^$^	448.3 ± 11.21 ^$^	4.48 ± 0.13^$^
PPA + SNL60+ DNP3	8.83 ± 0.41^$^	5.43 ± 0.23^$^	495.8 ± 4.43 ^$^	5.55 ± 0.13^$^
PPA + SNL60+ ARP5	10.07 ± 0.14 ^$β^	7.29 ± 0.23 ^$β^	539.7 ± 7.28 ^$β^	6.33 ± 0.08 ^$β^

Values expressed as mean ± SEM; * p < 0.001 versus sham control and SNL60 *perse*; **#** p < 0.001versus PPA; **#@** p < 0.001 versus PPA + SNL40; $ p < 0.001 versus PPA  + SNL60; $β p < 0.001 versus DNP3, MEM5, CTP10 (one way ANOVA followed by Tukey’s test).

### 3.4 Neuroprotective effect of SNL in neurotransmitter assessments ICV-PPA treated autistic rats

Neurotransmitter levels in the chronic administration of toxin PPA in rats showed a significant decrease (p < 0.001) in dopamine, serotonin, acetylcholine, and an increase in glutamate concentration in brain homogenate. After chronic treatment with SNL 40 and 60 mg/kg alone and in combination with standard drugs MEM 5 mg/kg, DNP 3 mg/kg, CTP 10 mg/kg and ARP 5 mg/kg remarkable (p < 0.001) increase in dopamine, acetylcholine and decreased glutamate seen. Effective restoration in neurotransmitter level of Ach seen in SNL 40 and 60 mg/kg when given in combination with DNP 3 mg/kg, whereas with serotonin influential restoration observed in SNL 40 and 60 mg/kg along with CTP 10 mg/kg. In other neurotransmitters such as dopamine effective restoration seen in SNL 40 and 60 mg/kg in combination with ARP 5 mg/kg, whereas glutamate level influentially decreased in MEM 5 mg/kg (Table 3).

**Table 3:** Neuroprotective Effect of SNL on neurotransmitters level (serotonin, Dopamine, Glutamate, acetylcholine) estimations in PPA treated rats.

Neurotransmitter levels							
Grouping	5-HT^a^ (ng/mg protein)	Grouping	Glutamate^b^ (ng/mg protein)	Grouping	Dopamine^c^ (ng/mg protein)	Grouping	Ach^d^ (ng/mg protein)
Sham control	32.00 ± 0.85	Sham control	98.17 ± 0.79	Sham control	82.00 ± 0.57	Sham control	5.44 ± 0.04
SNL *perse*	31.33 ± 0.66	SNL *perse*	96.33 ± 0.49	SNL *perse*	80.50 ± 0.76	SNL *perse*	5.24 ± 0.02
PPA 4 μl/0.26 M	7.83 ± 0.30*	PPA 4 μl/0.26 M	273.30 ± 0.91*	PPA 4 μl/0.26 M	24.17 ± 0.60*	PPA 4 μl/0.26 M	0.32 ± 0.01*
PPA + SNL40	10.83 ± 0.30^#^	PPA + SNL40	196.20 ± 2.35^#^	PPA + SNL40	39.50 ± 0.99^#^	PPA + SNL40	0.69 ± 0.04^#^
PPA + SNL60	13.67 ± 0.21^#@^	PPA + SNL60	167.00 ± 0.73^#@^	PPA + SNL60	43.67 ± 1.05^#@^	PPA + SNL60	0.94 ± 0.01^#@^
PPA + SNL60 + DNP3	20.00 ± 0.44 ^$^	PPA + SNL + CTP10	147.20 ± 1.10 ^$^	PPA + SNL60 + DNP3	58.50 ± 0.42 ^$^	PPA + SNL + CTP10	1.93 ± 0.01^$^
PPA + SNL60 + MEM5	23.50 ± 0.42 ^$^	PPA + SNL60+ DNP3	127.00 ± 1.23 ^$^	PPA + SNL60 + CTP10	62.17 ± 0.70 ^$^	PPA + SNL60 + ARP5	2.82 ± 0.04^$^
PPA + SNL60+ ARP5	27.33 ± 0.33 ^$^	PPA + SNL60+ ARP5	115.70 ± 2.66 ^$^	PPA + SL60 + MEM5	66.00 ± 0.57 ^$^	PPA + SNL60 + MEM5	3.70 ± 0.09^$^
PPA + SNL60 + CTP10	31.00 ± 0.57 ^$β^	PPA + SNL60 + MEM5	105.50 ± 1.45 ^$β^	PPA + SNL60 + ARP5	73.83 ± 0.94 ^$β^	PPA + SNL60 + DNP3	4.08 ± 0.02 ^$β^

a Values expressed as mean ± SEM; * p < 0.001 versus normal control and SNL60 *perse;***#**p < 0.001 versus PPA; **#@** p < 0.001 versus PPA + SNL40; $ p < 0.05 versus PPA + SNL60; $β p < 0.001 versus ARP5, MEM5, DNP3 (one way ANOVA followed by Tukey’s test).

b Values expressed as mean ± SEM; * p < 0.001 versus normal control and SNL60 *perse;***#**p < 0.001 versus PPA; **#@** p < 0.001 versus PPA + SNL40; $ p < 0.05 versus PPA + SNL60; $β p < 0.001 versus ARP5, DNP3, CTP10 (one way ANOVA followed by Tukey’s test).

c Values expressed as mean ± SEM; * p < 0.001 versus normal control and SNL60 *perse;***#**p < 0.001 versus PPA; **#@** p < 0.001 versus PPA + SNL40; $ p < 0.05 versus PPA + SNL60; $β p < 0.001 versus MEM5, ARP5, CTP10 (one way ANOVA followed by Tukey’s test).

d Values were expressed as mean ± SEM; * p < 0.001 versus normal control and SNL60 *perse;***#**p < 0.001 versus PPA; **#@** p < 0.001 versus PPA + SNL40; $ p < 0.05 versus PPA + SNL60; $β p < 0.001 versus MEM5, ARP5, CTP10 (one way ANOVA followed by Tukey’s test).

### 3.5 Neuroprotective effect of SNL on neuroinflammatory markers (TNF-α and IL-1β estimation in ICV-PPA treated autistic rats

The chronic administration of toxin PPA in rats showed a significant increase (p < 0.001) in pro-inflammatory cytokines such as TNF-α and IL-1β. After chronic treatment with SNL 40 and 60 mg/kg alone and in combination with standard drugs MEM 5 mg/kg, DNP 3 mg/kg, CTP 10 mg/kg and ARP 5 mg/kg had shown remarkable (p < 0.001) decrease in pro-inflammatory cytokines. The most significant decrease in TNF-α and IL-1β seen in SNL 40 and 60 mg/kg in combination with ARP 5 mg/kg (Table 4).

**Table 4:** Neuroprotective Effect of SNL on pro-inflammatory cytokines in ICV-PPA treated autistic rats.

Grouping	Neuro-inflammatory markers	
	TNF-α (pg/mg protein)	IL-1β (pg/mg protein)
Sham control	23.83 ± 0.79	7.50 ± 0.42
SNL *perse*	23.50 ± 0.76	7.33 ± 0.49
PPA 4 μl/0.26 M	61.33 ± 0.76*	17.17 ± 0.79*
PPA + SNL40	55.67 ± 1.02^#^	15.83 ± 0.30^#^
PPA + SNL60	43.00 ± 0.85^#@^	15.50 ± 0.22^#@- *^
PPA + SNL + CTP10	54.00 ± 0.93 ^$^	14.17 ± 0.40 ^$^
PPA + SL60+MEM5	52.83 ± 1.07 ^$^	13.83 ± 0.30 ^$^
PPA + SNL60+DNP3	43.50 ± 1.25 ^$^	12.83 ± 0.47 ^$^
PPA + SNL60+ARP5	35.00 ± 1.31 ^$β^	10.50 ± 0.42 ^$β^

Values were expressed as mean ± SEM; * p < 0.001 versus normal control and SNL60 *perse;***#**p < 0.001 versus PPA; **#@** p < 0.001 versus PPA + SNL40; $ p < 0.05 versus PPA + SNL60; $β p < 0.001 versus DNP3, MEM5, CTP10 (One way ANOVA followed by Tukey’s test).

### 3.6 Neuroprotective effect of SNL on oxidative stress markers (AchE, GSH, LDH, MDA, nitrite, and SOD) in ICV-PPA treated autistic rats

Neurochemical levels of AchE, MDA, LDH, and nitrite were significantly (p < 0.0001) increase, whereas levels of reduced GSH and SOD were significantly (p < 0.0001) decrease in ICV-PPA treated rats as compared to sham control and SNL 60 mg/kg. Chronic treatment with SNL 40 and 60 mg/kg alone and along with MEM 5 mg/kg, DNP 3 mg/kg, CTP 10 mg/kg and ARP 5 mg/kg progressively decrease in levels of AchE, MDA, LDH and nitrite and remarkably restore the anti-oxidant levels of reduced GSH and SOD. Among all groups, DNP 3 mg/kg given in continuation with SNL 60 mg/kg showed a profound decrease (p < 0.0001) in AchE, MDA, LDH and nitrite levels whereas, progressive restoration (p < 0.0001) found in levels of GSH and SOD when compared with all other interventional groups (Table 5).

**Table 5:** Neuroprotective effect of SNL on oxidative stress markers (AchE, GSH, LDH, MDA, nirite and SOD) in ICV-PPA treated autistic rats.

Oxidative stress markers						
Groups	AchE (μM/mg protein)	GSH (μM/mg protein)	LDH (Unit/mg protein)	MDA (nM/mg protein)	Nitrite (μM/mg protein)	SOD (μM/mg protein)
Sham control	11.67 ± 0.17	24.78 ± 0.11	99.33 ± 0.33	23.83 ± 0.79	2.56 ± 0.07	448.3 ± 0.66
SNL *perse*	11.58 ± 0.20	25.±0.15	98.00 ± 0.36	23.50 ± 0.76	2.56 ± 0.42	447.2 ± 0.94
PPA 4 μl/0.26 M	43.83 ± 1.17*	1.2 ± 0.01*	372.80 ± 0.87*	61.33 ± 0.76*	3.71 ± 0.01*	305.7 ± 1.68*
PPA + SNL40	30.33 ± 0.21^#^	2.65 ± 0.17^#^	277.00 ± 0.57^#^	55.17 ± 0.47^#^	3.61 ± 0.01^#^	318.3 ± 0.88^#^
PPA + SNL60	23.67 ± 0.21^#@^	3.73 ± 0.08^#@^	252.00 ± 0.85^@^	48.33 ± 0.88^#@^	3.52 ± 0.01^#@^	335.3 ± 0.80^#@^
PPA + SNL60+ CTP10	21.67 ± 0.21 ^$^	5.65 ± 0.07^$^	198.70 ± 0.49 ^$^	39.50 ± 0.42 ^$^	3.23 ± 0.01^$^	349.7 ± 0.66 ^$^
PPA + SNL60+ ARP5	19.50 ± 0.22 ^$^	7.58 ± 0.09^$^	149.00 ± 0.51 ^$^	36.17 ± 0.60 ^$^	3.14 ± 0.01^$^	369.0 ± 0.73 ^$^
PPA + SL60+ MEM5	17.50 ± 0.22 ^$^	9.45 ± 0.10^$^	121.30 ± 0.80 ^$^	31.83 ± 0.60 ^$^	3.05 ± 0.01^$^	388.5 ± 0.88 ^$^
PPA + SNL60+ DNP3	13.67 ± 0.14 ^$β^	10.83 ± 0.16 ^$β^	117.70 ± 1.47 ^$β^	28.67 ± 0.49 ^$β^	2.80 ± 0.01 ^$β^	417.7 ± 1.68 ^$β^

Values were expressed as mean ± SEM; * p < 0.001 versus normal control and SNL60 *perse;***#**p < 0.001 versus PPA; **#@** p < 0.001 versus PPA + SNL40; $ p < 0.05 versus PPA + SNL60; $β p < 0.001 versus MEM5, ARP5, CTP10. (One way ANOVA followed by Tukey’s test).

## 4 Discussion

SNL is a precursor of CoQ10 and a critical intermediate in the synthesis of metabolically active Quinones, such as CoQ10.SNL shown to improve mitochondrial dysfunction. As per our literature survey, the neuroprotective action of Coenzyme Q10 showed a major role in the treatment of migraine, Parkinson’s disease, and neurodegenerative diseases [52,55]. The present study planned to investigate the neuroprotective role of SNL against ICV-PPA induced autistic rats in combination with standard drugs ARP, MEM, DNP, CTP to check influence on the efficacy of treatment with already clinically available therapy. In 2009 the FDA approves, Aripiprazole; An atypical antipsychotic for autistic kids aged 6- 17 years as a treatment for autism. Aripiprazole is used to provide relief from hyperexcitability [28]. SSRI Citalopram is an alternative antidepressant agent used to treat depression-like symptoms, clinically and pre-clinically ([33], Bezchlibnyk et al., 2000, [75]), Memantine, NMDA receptor antagonist used for irritative behaviour and shows anti-excitatory activity [86]; and Donepezil; act as a cognitive enhancer used for memory and cognitive dysfunctions in autistic patients [6,24].

ICV-PPA is one of the widely used and well established experimental models for autism. ICV treatment of PPA (endogenous to the human body) capable of inducing many behavioral and neuropathological changes in rats compatible with those remarked in autism. Autistic rats reported an impairment of social behaviour, typical cognitive disability, repetitive behaviour, and object-directed behaviour. Along with this, there is a marked increase in stress markers and neuroinflammation. Increased PPA levels in the brain may interfere with overall cellular metabolism via the uncoupling of mitochondrial function and direct inhibition of oxidative phosphorylation [85].

In the present study, we examined the neuroprotective effect of SNL in the restoration of the enzyme level of mitochondrial ETC-complexes. Current results reveal that there was a significant decrease in mitochondrial ETC-complexes in ICV-PPA induced autistic rats. Upon chronic treatment with SNL 60 mg/kg (p.o.) along with ARP restoration found in the level of mitochondrial ETC-complexes enzymes.

ICV-PPA, also known to cause spatial memory loss, according to previous reports. There was no change in memory seen on the 1 st day of the experiment schedule. After the 12th day upon chronic administration of ICV-PPA, the significant increase in ELT and a decrease in TSTQ observed which show a severe memory loss. On chronic treatment with SNL 60 mg/kg (p.o.) and in combination with standard drugs, DNP exhibits a significant increase in TSTQ and the decrease in ELT showing the restoration in memory loss [36,93].

Upon administration of ICV-PPA, locomotion remarkably decreased in the actophotometer of ICV-PPA induced autistic rats as similarly shown in previous research. Chronic treatment with SNL 60 mg/kg (p.o.) in combination with MEM shows marked enhancement in locomotion [9].

In the current study, neuromuscular coordination recorded using a beam crossing task. An increase in the number of slips shows that there is an impairment in muscle coordination in ICV-PPA injected autistic rats, whereas on chronic treatment with SNL 60 mg/kg (p.o.) in combination with CTP showed improvement in muscle coordination by decreasing the number of slips.

Immobility and mobility time observed in the forced swim test to check the depressive behavior of autistic rats. ICV-PPA rats had shown enhancement in immobility time and the decrease in mobility time showing rat’s depressive behavior. Upon chronic treatment with SNL at high dose 60 mg/kg (p.o.)along with MEM shows a marked increase in mobility time and a decrease in immobility time [14].

Neurotransmitter assessments performed to check the imbalance in neurotransmitter levels. ICV-PPA induced autistic rats had shown a decreased level of dopamine, serotonin, and acetylcholine whereas increases in the levels of glutamate as stated in previous research showing neuronal excitotoxicity. Long - term administration of SNL 60 mg/kg (p.o.)in combination with ARP (dopamine), CTP (serotonin), DNP (acetylcholine), and MEM (glutamate) restores neurotransmitter levels [76,95,96].

ICV-PPA rats showed that their increased levels of pro-inflammatory cytokines TNF-α and IL-1β showing neuronal inflammation. SNL 60 mg/kg (p.o.) along with ARP shows the protective action against neuroinflammation by decreasing the levels of these major inflammatory cytokines [47].

ICV administration of toxin PPA in rats showed a significant increase in oxidative stress markers such as MDA, Nitrite, LDH and AchE levels increased, whereas the level of anti-oxidant, levels of GSH, SOD concentration decreased. On chronic treatment with SNL 60 mg/kg (p.o.)alone and in combination with standard drugs, DNP restores the level of anti-oxidant and signifies that there is a decrease in oxidative stress markers [27,97].

## 5 Conclusion

Conclusively, the present study shows that ICV-PPA significantly enhances the impairment of spatial and working memory and biochemical alterations like neuroinflammatory and neurotransmitter imbalance. While, chronic treatment of SNL alone and in combination with standard drugs Aripiprazole, citalopram, donepezil, and memantine showing no side effect significantly improve the cognitive deficits, biochemical alterations along with reducing the level of oxidative stress. Therefore, the above findings suggested that SNL may be a neuroprotective approach against ICV-PPA induced autistic rats and expected to be an effective therapeutic agent for preventive treatment of autism.

## Declaration of Competing Interest

The authors have no conflict of interest to report.

## References

[1] Qiu S., Li Y., Li Y., Zhong W., Shi M., Zhao Q., Zhang K., Wang Y., Lu M., Zhu X., Jiang H. (2018). Association between SHANK3 polymorphisms and susceptibility to autism spectrum disorder. Gene.

[2] Hossain S., Abdalla A.M., Jamain S.N.B., Zaini J.H., Azad A.K. (2017). A review on proton-conducting electrolytes for clean energy and intermediate temperature-solid oxide fuel cells. Renewable and Sustainable Energy Reviews.

[3] Port R.G., Edgar J.C., Ku M., Bloy L., Murray R., Blaskey L., Levy S.E., Roberts T.P. (2016). Maturation of auditory neural processes in autism spectrum disorder—A longitudinal MEG study. NeuroImage: Clinical.

[4] Schopler E., Mesibov G.B. (2013). Learning and cognition in autism.

[5] Shen L., Lin Y., Sun Z., Yuan X., Chen L., Shen B. (2016). Knowledge-guided bioinformatics model for identifying autism spectrum disorder diagnostic MicroRNA biomarkers. Scientific reports.

[6] Schwalfenberg G., Rodushkin I., Genuis S.J. (2018). Heavy metal contamination of prenatal vitamins. Toxicology reports.

[7] Phillips J.A., McGrew S.G., Vanderbilt University (2002). Modulation of in vivo glutamine and glycine levels in the treatment of autism.

[8] Vargas R., Ponce-Canchihuamán J. (2017). Emerging various environmental threats to brain and overview of surveillance system with zebrafish model. Toxicology reports.

[9] Pescosolido M.F., Yang U., Sabbagh M., Morrow E.M. (2012). Lighting a path: genetic studies pinpoint neurodevelopmental mechanisms in autism and related disorders. Dialogues in clinical neuroscience.

[10] Sweeten T.L., Croen L.A., Windham G.C., Odell J.D., Stubbs E.G., Torres A.R. (2019). Brief Report: Low Rates of Herpesvirus Detection in Blood of Individuals with Autism Spectrum Disorder and Controls. Journal of autism and developmental disorders.

[11] Lozano V.L., Defarge N., Rocque L.M., Mesnage R., Hennequin D., Cassier R., de Vendômois J.S., Panoff J.M., Séralini G.E., Amiel C. (2018). Sex-dependent impact of Roundup on the rat gut microbiome. Toxicology reports.

[12] Muck R.E., Nadeau E.M.G., McAllister T.A., Contreras- Govea F.E., Santos M.C., Kung L. (2018). Silage review: Recent advances and future uses of silage additives. Journal of Dairy Science.

[13] Thomas R.H., Foley K.A., Mepham J.R., Tichenoff L.J., Possmayer F., MacFabe D.F. (2010). Altered brain phospholipid and acylcarnitine profiles in propionic acid infused rodents: further development of a potential model of autism spectrum disorders. Journal of neurochemistry.

[14] MacFabe D.F. (2012). Short-chain fatty acid fermentation products of the gut microbiome: implications in autism spectrum disorders. Microbial ecology in health and disease.

[15] Duberley K.E., Abramov A.Y., Chalasani A., Heales S.J., Rahman S., Hargreaves I.P. (2013). Human neuronal coenzyme Q10 deficiency results in global loss of mitochondrial respiratory chain activity increased mitochondrial oxidative stress and reversal of ATP synthase activity: implications for pathogenesis and treatment. Journal of Inherited Metabolic Disease: Official Journal of the Society for the Study of Inborn Errors of Metabolism.

[16] Mehan S., Monga V., Rani M., Dudi R., Ghimire K. (2018). Neuroprotective effect of solanesol against 3- nitropropionic acid-induced Huntington’s disease-like behavioral, biochemical, and cellular alterations: Restoration of coenzyme-Q10-mediated mitochondrial dysfunction. Indian journal of pharmacology.

[17] Ali S.H.K., Raja W.A. (2019). The effect of antioxidants in acute amitriptyline poisoning. Toxicology reports.

[18] Uttara B., Singh A.V., Zamboni P., Mahajan R.T. (2009). Oxidative stress and neurodegenerative diseases: a review of upstream and downstream antioxidant therapeutic options. Current neuropharmacology.

[19] Crane F.L., Löw H., Sun I., Navas P., Gvozdjáková A. (2014). Plasma membrane coenzyme Q: evidence for a role in autism. Biologics: targets & therapy.

[20] Goines P.E., Ashwood P. (2013). Cytokine dysregulation in autism spectrum disorders (ASD): possible role of the environment. Neurotoxicology and teratology.

[21] Owen R., Sikich L., Marcus R.N., Corey-Lisle P., Manos G., McQuade R.D., Carson W.H., Findling R.L. (2009). Aripiprazole in the treatment of irritability in children and adolescents with autistic disorder. Pediatrics.

[22] Chez M.G., Burton Q., Dowling T., Chang M., Khanna P., Kramer C. (2007). Memantine as adjunctive therapy in children diagnosed with autistic spectrum disorders: an observation of initial clinical response and maintenance tolerability. Journal of Child Neurology.

[23] Hardan A.Y., Handen B.L. (2002). A retrospective open trial of adjunctive donepezil in children and adolescents with autistic disorder. Journal of Child and Adolescent Psychopharmacology.

[24] King B.H., Hollander E., Sikich L., McCracken J.T., Scahill L., Bregman J.D., Donnelly C.L., Anagnostou E., Dukes K., Sullivan L., Hirtz D. (2009). Lack of efficacy of citalopram in children with autism spectrum disorders and high levels of repetitive behavior: citalopram ineffective in children with autism. Archives of general psychiatry.

[25] Blankenship K., Erickson C.A., Stigler K.A., Posey D.J., McDougle C.J. (2010). Aripiprazole for irritability associated with autistic disorder in children and adolescents aged 6–17 years. Pediatric health.

[26] LeClerc S., Easley D. (2015). Pharmacological therapies for autism spectrum disorder: a review. Pharmacy and Therapeutics.

[27] Farmer C.A., Aman M.G. (2011). Aripiprazole for the treatment of irritability associated with autism. Expert opinion on pharmacotherapy.

[28] Burke S.P., Stratton K., Baciu A. (2007). The future of drug safety: promoting and protecting the health of the public.

[29] Erickson C.A., Stigler K.A., Posey D.J., McDougle C.J. (2010). Aripiprazole in autism spectrum disorders and fragile X syndrome. Neurotherapeutics.

[30] Nirogi R., Kandikere V., Jayarajan P., Bhyrapuneni G., Saralaya R., Muddana N., Abraham R. (2013). Aripiprazole in an animal model of chronic alcohol consumption and dopamine D2 receptor occupancy in rats. The American journal of drug and alcohol abuse.

[31] Nowakowska E., Kus K., Ratajczak P., Cichocki M., Woźniak A. (2014). The influence of aripiprazole, olanzapine and enriched environment on depressant-like behavior, spatial memory dysfunction and hippocampal level of BDNF in prenatally stressed rats. Pharmacological Reports.

[32] Procyshyn R.M., Bezchlibnyk- Butler K.Z., Jeffries J.J. (2017). Clinical handbook of psychotropic drugs.

[33] Armenteros J.L., Lewis J.E. (2002). Citalopram treatment for impulsive aggression in children and adolescents: an open pilot study. Journal of the American Academy of Child & Adolescent Psychiatry.

[34] McPheeters M.L., Warren Z., Sathe N., Bruzek J.L., Krishnaswami S., Jerome R.N., Veenstra- VanderWeele J. (2011). A systematic review of medical treatments for children with autism spectrum disorders. Pediatrics..

[35] Verhoeven W.M., Veendrik- Meekes M.J., Jacobs G.A., Van Den Berg Y.W., Tuinier S. (2001). Citalopram in mentally retarded patients with depression: a long-term clinical investigation. European Psychiatry.

[36] Benvenuto A., Battan B., Porfirio M.C., Curatolo P. (2013). Pharmacotherapy of autism spectrum disorders. Brain and Development.

[37] Rodriguez‐Porcel F., Green D., Khatri N., Harris S.S., May W.L., Lin R.C., Paul I.A. (2011). Neonatal exposure of rats to antidepressants affects behavioral reactions to novelty and social interactions in a manner analogous to autistic spectrum disorders. The Anatomical Record..

[38] Bezchlibnyk-Butler K., Aleksic I., Kennedy S.H. (2000). Citalopram--a review of pharmacological and clinical effects. Journal of Psychiatry and Neuroscience..

[39] Nutt D.J. (2005). Overview of diagnosis and drug treatments of anxiety disorders. CNS spectrums.

[40] Montgomery S.A., Loft H., Sánchez C., Reines E.H., Papp M. (2001). Escitalopram (S‐enantiomer of citalopram): clinical efficacy and onset of action predicted from a rat model. Pharmacology & toxicology.

[41] Njung’e K., Handley S.L. (1991). Effects of 5‐HT uptake inhibitors, agonists and antagonists on the burying of harmless objects by mice; a putative test for anxiolytic agents. British journal of pharmacology..

[42] Thomas S.J., Grossberg G.T. (2009). Memantine: a review of studies into its safety and efficacy in treating Alzheimer’s disease and other dementias. Clinical interventions in aging.

[43] Zdanys K., Tampi R.R. (2008). A systematic review of off-label uses of memantine for psychiatric disorders. Progress in Neuro- Psychopharmacology and Biological Psychiatry.

[44] Findling R.L., McNamara N.K., Stansbrey R.J., Maxhimer R., Periclou A., Mann A., Graham S.M. (2007). A pilot evaluation of the safety, tolerability, pharmacokinetics, and effectiveness of memantine in pediatric patients with attention-deficit/hyperactivity disorder combined type. Journal of child and adolescent psychopharmacology..

[45] Erickson C.A., Early M., Stigler K.A., Wink L.K., Mullett J.E., McDougle C.J. (2011). An open-label naturalistic pilot study of acamprosate in youth with autistic disorder. Journal of child and adolescent psychopharmacology.

[46] Aman M.G., Findling R.L., Hardan A.Y., Hendren R.L., Melmed R.D., Kehinde-Nelson O., Hsu H.A., Trugman J.M., Palmer R.H., Graham S.M., Gage A.T. (2017). Safety and efficacy of memantine in children with autism: Randomized, placebo-controlled study and open- label extension. Journal of child and adolescent psychopharmacology.

[47] Minkeviciene R., Banerjee P., Tanila H. (2004). Memantine improves spatial learning in a transgenic mouse model of Alzheimer’s disease. Journal of Pharmacology and Experimental Therapeutics.

[48] Réus G.Z., Stringari R.B., Kirsch T.R., Fries G.R., Kapczinski F., Roesler R., Quevedo J. (2010). Neurochemical and behavioural effects of acute and chronic memantine administration in rats: further support for NMDA as a new pharmacological target for the treatment of depression?. Brain research bulletin.

[49] Zoladz P.R., Campbell A.M., Park C.R., Schaefer D., Danysz W., Diamond D.M. (2006). Enhancement of long-term spatial memory in adult rats by the noncompetitive NMDA receptor antagonists, memantine and neramexane. Pharmacology Biochemistry and Behavior..

[50] Parsons C.G., Danysz W., Quack G. (1999). Memantine is a clinically well tolerated N-methyl-D-aspartate (NMDA) receptor antagonist—a review of preclinical data. Neuropharmacology..

[51] Basselin M., Nguyen H.N., Chang L., Bell J.M., Rapoport S.I. (2009). Acute but not chronic donepezil increases muscarinic receptor-mediated signaling via arachidonic acid in unanesthetized rats. Journal of Alzheimer’s Disease..

[52] Burt T., Sachs G.S., Demopulos C. (1999). Donepezil in treatment-resistant bipolar disorder. Biological psychiatry.

[53] Buckley A.W., Sassower K., Rodriguez A.J., Jennison K., Wingert K., Buckley J., Thurm A., Sato S., Swedo S. (2011). An open label trial of donepezil for enhancement of rapid eye movement sleep in young children with autism spectrum disorders. Journal of child and adolescent psychopharmacology.

[54] Handen B.L., Johnson C.R., McAuliffe-Bellin S., Murray P.J., Hardan A.Y. (2011). Safety and efficacy of donepezil in children and adolescents with autism: neuropsychological measures. Journal of Child and Adolescent Psychopharmacology.

[55] Burns A., Rossor M., Hecker J., Gauthier S., Petit H., Möller H.J., Rogers S.L., Friedhoff L.T. (1999). The Effects of Donepezil in Alzheimer’s Disease–Results from a Multinational Trial1. Dementia and geriatric cognitive disorders.

[56] Kim J.W., Seung H., Kwon K.J., Ko M.J., Lee E.J., Oh H.A., Choi C.S., Kim K.C., Gonzales E.L., You J.S., Choi D.H. (2014). Subchronic treatment of donepezil rescues impaired social, hyperactive, and stereotypic behavior in valproic acid-induced animal model of autism. PloS one.

[57] Yoo J.H., Valdovinos M.G., Williams D.C. (2007). Relevance of donepezil in enhancing learning and memory in special populations: a review of the literature. Journal of autism and developmental disorders.

[58] Nirogi R., Daripelli S., Benade V., Tirumalasetty C., Bhyrapuneni G., Jayarajan P. (2017). Simultaneous monitoring of electroencephalographic characteristics in animals subjected to behavioral tests: a preclinical investigation. Behavioural pharmacology.

[59] Tsai C.J., Yu Y.H., Chiu H.J., Loh E.W., Wang J.T., Chan C.H., Lan T.H. (2011). The quantitative detection of aripiprazole and its main metabolite by using capillary-electrophoresis. Journal of the Chinese Medical Association.

[60] Kanemaru K., Hasegawa S., Nishi K., Diksic M. (2008). Acute citalopram has different effects on regional 5-HT synthesis in FSL, FRL, and SDP rats: an autoradiographic evaluation. Brain research bulletin.

[61] Al Asmari A.K., Ullah Z., Tariq M., Fatani A. (2016). Preparation, characterization, and in vivo evaluation of intranasally administered liposomal formulation of donepezil. Drug design, development, and therapy.

[62] Landa L., Slais K., Sulcova A. (2012). The effect of memantine on behavioural sensitization to methamphetamine in mice. Veterinární medicína.

[63] Thomas A., Lee P.J., Dalton J.E., Nomie K.J., Stoica L., Costa-Mattioli M., Chang P., Nuzhdin S., Arbeitman M.N., Dierick H.A. (2012). A Versatile Method for Cell-Specific Profiling of Translated mRNAs in Drosophila. PLoS ONE.

[64] Barai A., Uddin K., Widanage W.D., McGordon A., Jennings P. (2018). A study of the influence of measurement timescale on internal resistance characterization methodologies for lithium-ion cells. Scientific reports.

[65] Lü J.M., Lin P.H., Yao Q., Chen C. (2010). Chemical and molecular mechanisms of antioxidants: experimental approaches and model systems. Journal of cellular and molecular medicine.

[66] Choi J., Lee S., Won J., Jin Y., Hong Y., Hur T.Y., Kim J.H., Lee S.R., Hong Y. (2018). Pathophysiological and neurobehavioral characteristics of a propionic acid-mediated autism-like rat model. PloS one.

[67] Morris R. (1984). Developments of a water-maze procedure for studying spatial learning in the rat. Journal of Neuroscience Methods.

[68] Frye R.E., Nankova B., Bhattacharyya S., Rose S., Bennuri S.C., MacFabe D.F. (2017). Modulation of immunological pathways in autistic and neurotypical lymphoblastoid cell lines by the enteric microbiome metabolite propionic acid. Frontiers in Immunology.

[69] Shultz S.R., MacFabe D.F., Martin S., Jackson J., Taylor R., Boon F., Ossenkopp K.P., Cain D.P. (2009). Intracerebroventricular injections of the enteric bacterial metabolic product propionic acid impair cognition and sensorimotor ability in the Long–Evans rat: further development of a rodent model of autism. Behavioural brain research.

[70] Shultz S.R., Bao F., Omana V., Chiu C., Brown A., Cain D.P. (2012). Repeated mild lateral fluid percussion brain injury in the rat causes cumulative long-term behavioral impairments, neuroinflammation, and cortical loss in an animal model of repeated concussion. Journal of neurotrauma.

[71] Ignácio Z.M., Réus G.Z., Abelaira H.M., de Moura A.B., de Souza T.G., Matos D., Goldim M.P., Mathias K., Garbossa L., Petronilho F., Quevedo J. (2017). Acute and chronic treatment with quetiapine induces antidepressant-like behavior and exerts antioxidant effects in the rat brain. Metabolic brain disease.

[72] Jones L.L. (2009). Alterations in metabolism during ketogenic therapy for seizures.

[73] Höglinger G.U., Carrard G., Michel P.P., Medja F., Lombès A., Ruberg M., Friguet B., Hirsch E.C. (2003). Dysfunction of mitochondrial complex I and the proteasome: interactions between two biochemical deficits in a cellular model of Parkinson’s disease. Journal of neurochemistry.

[74] Bhateja D.K., Dhull D.K., Gill A., Sidhu A., Sharma S., Reddy B.K., Padi S.S. (2012). Peroxisome proliferator-activated receptor-α activation attenuates 3-nitropropionic acid-induced behavioral and biochemical alterations in rats: possible neuroprotective mechanisms. European journal of pharmacology.

[75] Ramanathan R., Ramanathan U., Hsiao H.L. (2012). The impact of e-commerce on Taiwanese SMEs: Marketing and operations effects. International Journal of Production Economics.

[76] Takada M., Yuzuriha T., Yamato C. (1985). Redox levels of intravenously administered [14C] coenzyme Q10 and coenzyme Q10-reducing activity in subcellular fractions of guinea pig liver. Journal of nutritional science and vitaminology.

[77] Jamwal S., Singh S., Kaur N., Kumar P. (2015). Protective effect of spermidine against excitotoxic neuronal death induced by quinolinic acid in rats: possible neurotransmitters and neuroinflammatory mechanisms. Neurotoxicity Research.

[78] Patel B.A., Arundell M., Parker K.H., Yeoman M.S., O’Hare D. (2005). Simple and rapid determination of serotonin and catecholamines in biological tissue using high- performance liquid chromatography with electrochemical detection. Journal of Chromatography B.

[79] Ren J., Bai Y., Hao L., Dong Y., Pi Z., Jia L. (2011). Amelioration of experimental autoimmune myasthenia gravis rats by blood purification treatment using 4‐mercaptoethylpyridine‐based adsorbent. Journal of biomedical materials research Part A.

[80] Winkler G., Salamon F., Harmos G., Salamon D., Speer G., Szekeres O., Hajos P., Kovacs M., Simon K., Cseh K. (1998). Elevated serum tumor necrosis factor-alpha concentrations and bioactivity in Type 2 diabetics and patients with android type obesity. Diabetes research and clinical practice.

[81] Vedi M., Rasool M., Sabina E.P. (2014). Amelioration of bromobenzene hepatotoxicity by Withania somnifera pretreatment: role of mitochondrial oxidative stress. Toxicology reports.

[82] Rege K., Medintz I.L. (2009). Methods in Bioengineering: Nanoscale Bioengineering and Nanomedicine.

[83] El-Ansary A.K., Bacha A.B., Kotb M. (2012). Etiology of autistic features: the persisting neurotoxic effects of propionic acid. Journal of neuroinflammation.

[84] Sharma N., Gautam S., Devi U., Singh M., Rawat J.K., Sethi N., Saraf S.A., Kaithwas G. (2015). Preclinical appraisal of terbutaline analogues in precipitation of autism spectrum disorder. RSC Advances.

[85] Ellman G.L. (1959). Tissue sulfhydryl groups. Archives of Biochemistry and Biophysics.

[86] Bronson S.K., Plaehn E.G., Kluckman K.D., Hagaman J.R., Maeda N., Smithies O. (1996). Single-copy transgenic mice with chosen-site integration. Proceedings of the National Academy of Sciences of the United States of America.

[87] González-Fraguela M.E., Hung M.L.D., Vera H., Maragoto C., Noris E., Blanco L., Galvizu R., Robinson M. (2013). Oxidative stress markers in children with autism spectrum disorders. Journal of Advances in Medicine and Medical Research.

[88] Khalil S.R., Abd- Elhakim Y.M., Selim M.E., Al-Ayadhi L.Y. (2015). Apitoxin protects rat pups brain from propionic acid-induced oxidative stress: the expression pattern of Bcl-2 and Caspase-3 apoptotic genes. Neurotoxicology.

[89] Bhandari R., Kuhad A. (2015). Neuropsychopharmacotherapeutic efficacy of curcumin in the experimental paradigm of autism spectrum disorders. Life sciences.

[90] Morris G., Anderson G., Berk M., Maes M. (2013). Coenzyme Q10 depletion in medical and neuropsychiatric disorders: potential repercussions and therapeutic implications. Molecular neurobiology.

[91] Emerit J., Edeas M., Bricaire F. (2004). Neurodegenerative diseases and oxidative stress. Biomedicine & pharmacotherapy.

[92] Tania M., Khan M.A., Xia K. (2014). Recent advances in animal model experimentation in autism research. Acta neuropsychiatrica.

[93] Holbrook S. (2013). The effects of a single acute and repeated intracerebroventricular infusions of propionic acid on locomotor activity and neuroinflammation in rats.

[94] Bumin G., Günal A., Tükel Ş. (2008). Anxiety, depression, and quality of life in mothers of disabled children. SDÜ Tıp Fak Derg.

[95] Rubenstein J.L.R., Merzenich M.M. (2003). Model of autism: increased ratio of excitation/inhibition in key neural systems. Genes, Brain and Behavior.

[96] Li X., Chauhan A., Sheikh A.M., Patil S., Chauhan V., Li X.M., Ji L., Brown T., Malik M. (2009). Elevated immune response in the brain of autistic patients. Journal of neuroimmunology.

[97] Farkhondeh T., Samarghandian S. (2016). Antidotal effects of curcumin against agents-induced cardiovascular toxicity. Cardiovascular & Haematological Disorders-Drug Targets (Formerly Current Drug Targets-Cardiovascular & Hematological Disorders).

